# AI-dente: an open machine learning based tool to interpret nano-indentation data of soft tissues and materials†

**DOI:** 10.1039/d3sm00402c

**Published:** 2023-08-25

**Authors:** Patrick Giolando, Sotirios Kakaletsis, Xuesong Zhang, Johannes Weickenmeier, Edward Castillo, Berkin Dortdivanlioglu, Manuel K. Rausch

**Affiliations:** a The University of Texas at Austin, Department of Biomedical Engineering USA; b The University of Texas at Austin, Department of Aerospace Engineering & Engineering Mechanics USA; c Stevens Institute of Technology, Department of Mechanical Engineering USA; d The University of Texas at Austin, Department of Civil, Environmental, and Architectural Engineering USA berkin@utexas.edu; e The University of Texas at Austin, Department of Mechanical Engineering USA; f The University of Texas at Austin, Oden Institute for Computational Engineering and Sciences USA

## Abstract

Nano-indentation is a promising method to identify the constitutive parameters of soft materials, including soft tissues. Especially when materials are very small and heterogeneous, nano-indentation allows mechanical interrogation where traditional methods may fail. However, because nano-indentation does not yield a homogeneous deformation field, interpreting the resulting load–displacement curves is non-trivial and most investigators resort to simplified approaches based on the Hertzian solution. Unfortunately, for small samples and large indentation depths, these solutions are inaccurate. We set out to use machine learning to provide an alternative strategy. We first used the finite element method to create a large synthetic data set. We then used these data to train neural networks to inversely identify material parameters from load–displacement curves. To this end, we took two different approaches. First, we learned the indentation forward problem, which we then applied within an iterative framework to identify material parameters. Second, we learned the inverse problem of directly identifying material parameters. We show that both approaches are effective at identifying the parameters of the neo-Hookean and Gent models. Specifically, when applied to synthetic data, our approaches are accurate even for small sample sizes and at deep indentation. Additionally, our approaches are fast, especially compared to the inverse finite element approach. Finally, our approaches worked on unseen experimental data from thin mouse brain samples. Here, our approaches proved robust to experimental noise across over 1000 samples. By providing open access to our data and code, we hope to support others that conduct nano-indentation on soft materials.

## Abstract 2

Machine learning can improve the identification of soft material parameters from nano-indentation experiments.

## Graphical abstract

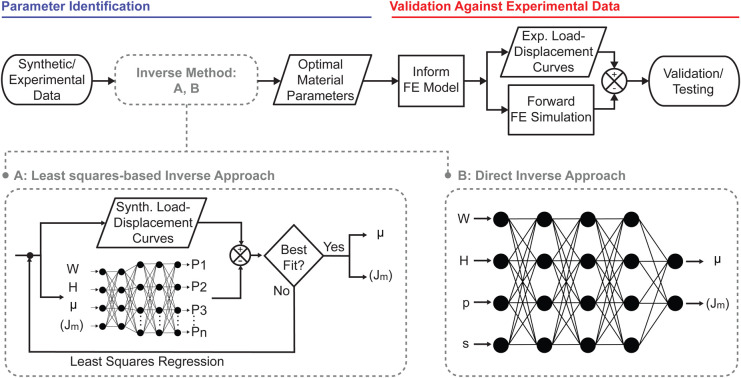

## 1 Introduction

Testing and quantifying the mechanical properties of soft materials, such as soft tissues, is essential to understanding and predicting their behavior.^1–3^ Unfortunately, the experimental characterization of soft materials faces numerous difficulties. This is especially true for soft tissues that are often heterogeneous as well as very small.^4–7^ Traditional test methods, such as uniaxial tensile-compression testing, may not capture the heterogeneity of the tissue, as these tests tend to interrogate only the bulk of the material and do not capture spatial variations.^8^ Moreover, traditional test methods often require significant extra sample space for clamping,^9^ which proves difficult or impossible when working with small biological samples. For example, when using model systems such as rodents, tissues’ lateral dimensions can be on the order of millimeters while having thicknesses on the order of micrometers.^10–14^

The inability of traditional test methods to yield spatially resolved mechanical properties and to accommodate very small test samples has inspired the use of indentation-based methods. Such methods may use micrometer-sized indenters ranging in size from a few to hundreds of micrometers that locally probe soft biological tissues to yield load–displacement curves.^15–18^ The qualitative and quantitative characteristics of these curves may then be interpreted to yield approximations for the local mechanical properties of the material of interest. Thus, these methods overcome the limitations of traditional mechanical test methods and can not only be applied to very small test samples without the need for mechanical clamping but can be repeated in a scanning pattern over the tissue to map the samples heterogeneity.^19–22^

However, indentation-based methods face their own challenges. Aside from experimental hurdles, indentation yields highly nonlinear deformations. To interpret these data, the community has largely resorted to using the spherical Hertzian contact solution.^23,24^ However, this solution has some limitations in that it applies only to linearly elastic isotropic materials, assumes that the indenter and sample surface are non-conforming, requires that the indentation depth is much smaller compared to the spherical indenter diameter, and finally, it ignores friction and surface effects.^25^ The indentation of soft materials, including biological soft tissues and hydrogels, in practice, violates at least some of these assumptions.^26,27^ Therefore, using the Hertzian model to identify the material parameters of soft biological tissues *via* nano-indentation is prone to significant errors.^28–32^

Previously developed approaches for overcoming some of the Hertzian model's pitfalls each introduce their own limitations. For example, Zhang *et al.* recently proposed a modification that corrects the Hertzian model for large indentations (>10% of the indenter radius).^33^ However, this solution, and other proposed modifications to the Hertzian model, usually only overcome one of its limitations, but not all. In contrast, others have used inverse finite element approaches to identify material parameters through an iterative least squares approach in which either the direct inverse problem or iterative forward problems are solved to identify unknown material parameters from indentation data.^34–38^ The flexibility of finite element methods allows these approaches to overcome all of the Hertzian model's limitations. However, inverse finite element approaches can be computationally expensive.^39,40^

The objective of our current work is to develop an efficient approach that combines the generality of finite element-based methods with the high computational efficiency of machine learning. Thereby, we will provide an open-source tool that identifies the material parameters of biological soft tissues – and other soft materials – from indentation data at a much lower cost than classic inverse finite element approaches. To this end, we use the finite element method to create two large synthetic data sets for the neo-Hookean and Gent models and subsequently use them to train neural networks that identify their material parameters from load–displacement data.

## 2 Methods

### 2.1 Synthetic data creation

To create synthetic data for model training, testing, and validation we first sampled a four- and five-dimensional parameter space for the nano-indentation problem with neo-Hookean and Gent models, respectively. The parameter space included sample width (*W*), sample thickness (*H*), indentation depth (*δ*), sample shear modulus (*μ*), and the Gent material parameter (*J*_m_), see Fig. 2. The indenter radius (*R*) was used as the characteristic length to non-dimensionalize all geometric parameters. The final parameter space spanned 5 ≤ *W*/*R* ≤ 40 by 5 ≤ *H*/*R* ≤ 40 by 0.05 ≤ *δ*/*R* ≤ 0.5 by 10^2^ Pa ≤ *μ* ≤ 10^6^ Pa and 5 × 10^−4^ ≤ *J*_m_ ≤ 5. We then sampled this large parameter space using latin hypercube sampling. Note that we logarithmically scaled the shear moduli before sampling. With each set of parameters, we created a finite element input file for the nonlinear finite element solver FEBio (www.febio.org). Within FEBio, we then synthetically simulated the nonlinear indentation problem of a rectangular prism of dimensions *W* × *W* × *H* to yield our training, validation, and testing load–displacement data sets. In total we generated 25 000 data sets: a 10 000 sample training set, 1250 validation set, and 1250 test data set for both, the neo-Hookean model and Gent model. Please note, as the name suggests, the training data was used for training our machine learning-based approaches, while the validation data was used in network selection and hyperparameter tuning. Finally, the testing data set was only used after training and parameter tuning were concluded, to test the success of our approach.

### 2.2 Machine learning based inverse approaches

**Fig. 1:**
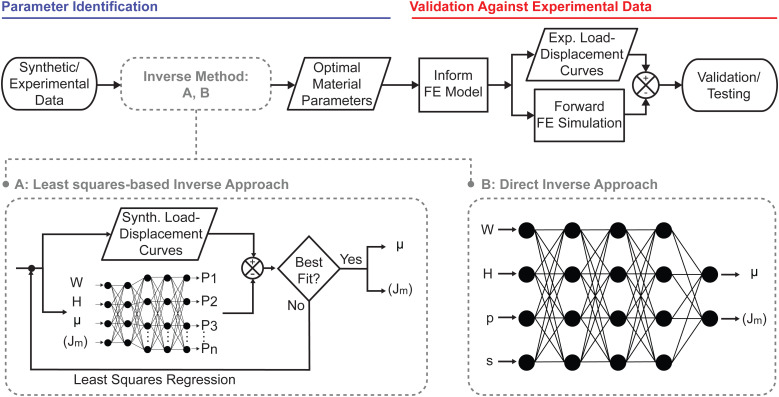
Inverse parameter identification and validation/testing procedures. After creating synthetic (Synth.) data sets, we trained a neural network to either solve the forward problem or to solve the direct inverse problem. That is, we trained a neural network to either predict load–displacement curves (depicted as discrete load points, *i.e.*, P1 through Pn) from geometric and material input parameters, or we trained a neural network to predict material parameters from load–displacement and geometric information. While the latter approach directly predicts material parameters from indentation experiments, the former approach must be combined with an iterative (in our case, least squares) approach. Once material parameters have been predicted using either method, we used them as inputs to a standard forward finite element simulation to output load–displacement predictions for validation against experimental (Exp.) data.

In this study we compared two different machine learning approaches to accelerate material parameter identification from indentation data, see Fig. 1. First, we used a least squares-based approach in which we trained a neural network to solve the forward problem, see Fig. 1(A). In the second approach, we trained a neural network to directly predict material parameters from load–displacement data, without the need of iterations, see Fig. 1(B).

For both the least squares and the direct inverse approach, we chose a fully connected dense neural network or multilayer perceptron. The hidden layer activation functions were set to leaky ReLU (*α* = 0.3), and the output layer activation function was set to linear. The neural network for the forward problem used an Adam optimizer and mean squared error as the loss metric to learn the mapping between *W*, *H*, *μ*, *J*_m_ and load–displacement data pairs sampled every 0.005*R* between *δ* = 0 and *δ* = 0.5*R*. The architecture for the forward neural network consisted of 5 hidden layers, the first two layers having 4 nodes and the last 3 layers having 100 nodes. Similarly, the neural network for the direct inverse problem used Adam as an optimizer and mean averaged error as the loss metric. Instead of using full load–displacement curves as input features, we parameterized the load–displacement curves by fitting them to a power law, *viz. F*(*δ*) = *pδ*^*s*^. In turn, we used *p* and *s*, together with the geometric parameters *W* and *H*, as the input features for the direct inverse approach. That is, we trained our second neural network to map *W*, *H*, *p*, *s* to the material parameters *μ* and *J*_m_. The architecture for the direct inverse neural network consisted of 5 hidden layers, the first four layers having 4 nodes and the last layer having 2 nodes.

### 2.3 Finite element model details

**Fig. 2:**
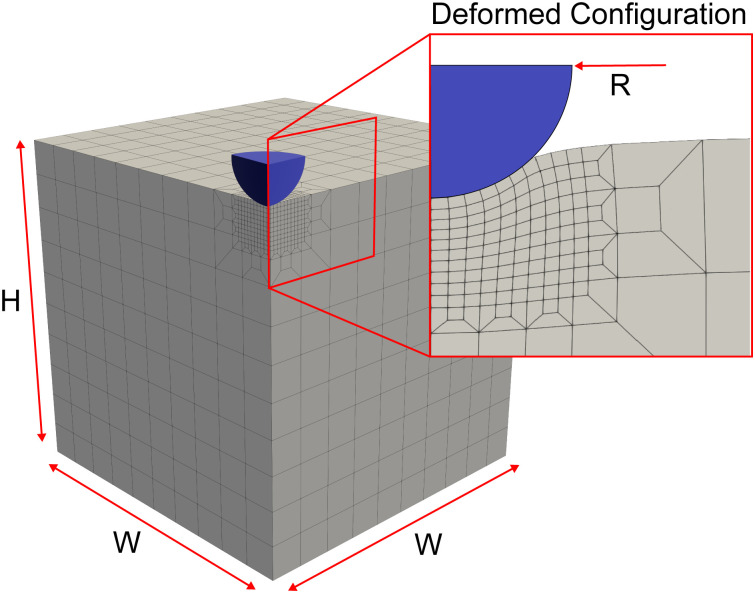
Illustration of the finite element domain and discretization for the indentation problem. The insert shows the deformed configuration after displacing the rigid (blue) indenter of radius *R* to contact the indented material (grey) of dimensions *W* × *W* × *H*.

We simulated the indentation problem using the nonlinear finite element solver FEBio (Version 3.0.0). To this end, we created a rigid sphere that was displaced by *δ* to indent a soft material domain of dimensions *W* × *W* × *H*, see Fig. 2. After careful convergence studies, we discretized the domain with a biased mesh with 1609 to 65 484 elements – depending on domain size – using mixed hexahedral elements.^41^ The contact itself was modeled as frictionless. We conducted these simulations for two hyperelastic material models, the neo-Hookean model and the Gent model.^42^ We chose the former for its popularity in the biomechanics community, but also because it yields a single material parameter that can be easily compared to other, common measures of material stiffness, such as Young's modulus. In contrast, we chose the Gent model for its ability to capture a wide spectrum of strain-stiffening material behaviors as may be seen during indentation experiments on soft tissues. The strain energy density functions for both models read1
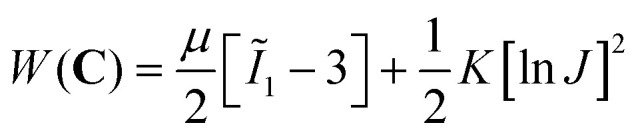
and2
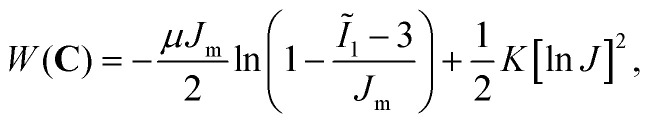
respectively. Here, *μ* is the shear modulus, *J*_m_ is the stiffening parameter for the first invariant, and *K* is the bulk modulus. In all of our simulations, we chose *K* to be three orders of magnitude larger than *μ* to ensure quasi-compressibility.^43^ Note that *Ĩ*_1_ is the first invariant of the isochoric Cauchy–Green deformation tensor and *J* is the determinant of the deformation gradient. More details are available in the FEBio documentation and in the relevant literature on hyperelastic constitutive modeling.^44^

### 2.4 Hertzian and modified Hertzian contact theory

The Hertzian solution was originally formulated as a simplified contact model for a rigid sphere and an elastic half-space. It relies on the assumption that the surface is an infinite half plane, the pressure distribution is parabolic, the material is homogeneous, and that the material strain is small. By integrating the pressure over the region under compression, a practical relationship between applied force (F) and vertical displacement (*δ*) of the indenter thus follows as3
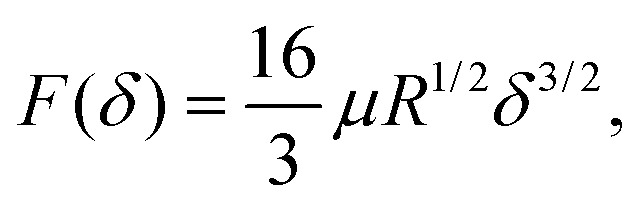
where *μ* is the shear modulus of the indented material.^45^ Please note that we assumed a perfectly rigid indenter material and an incompressible indented material to arrive at the above expression of the classic Hertzian formulation. When violating the small strain and the nonconformity of surfaces assumptions, indentation depth-dependent discrepancies between the Hertzian solution and finite element solutions have been reported.^46^ This has led to the development of numerous modified Hertzian solutions. Here we chose one for comparison to our approach,^33^*viz.*4
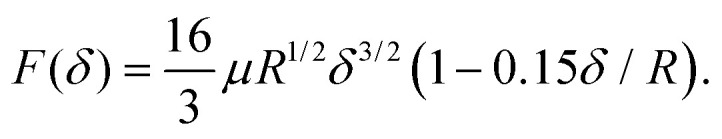


It is the last term in this modified solution that improves the standard Hertzian predictions for large indentation depths.

### 2.5 Real-world mouse brain indentation data

We used real-world data to test out our machine learning-based approach. That is, we collected 1372 load–displacement curves by indenting both fresh and fixed mouse brain tissue. Brains were harvested from two 14-week-old female C57BL/6 mice before using a vibratome (Leica Biosystems, Buffalo Grove, IL) to slice each brain into 1 mm thick samples. “Fixed” samples from animal #1 were first fixed for 24 hours in 10% neutral buffered formalin solution, while “fresh” samples from animal #2 were immediately transferred to our nano-indentation tester (FT-MTA03, FemtoTools AG, Switzerland). Indentation tests on fixed and fresh samples were then performed using a FT-S200 probe head with a 50 μm polystyrene bead. Note, the probe has a ±200 μN sensing range and a resolution of 0.0005 μN. We probed the entire sample surfaces at 75 μm inter-measurement spacing and an indentation speed of 10 μm s^−1^ for a total of 686 indentations in fresh tissue and 686 indentations in fixed tissue. All research and animal care procedures were approved by the Institutional Review Board at Stevens Institute of Technology under animal protocol 2019-004(AP) and performed according to international guidelines on the use of laboratory animals.

## 3 Results

### 3.1 Sensitivity of indentation

**Fig. 3:**
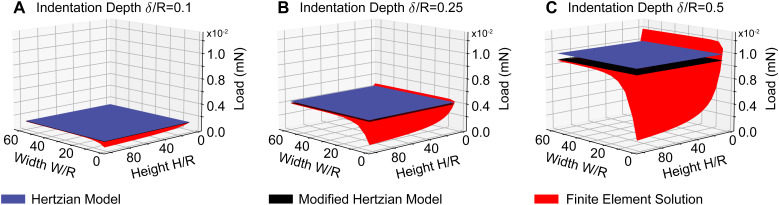
Comparison between the (modified) Hertzian approach and the gold-standard finite element solution. The predicted maximum load due to indentation to a depth of 0.1 (A), 0.25 (B), and 0.5 (C) times the indenter radius. With decreasing lateral size (*W*) and thickness (*H*) the Hertzian solution and its modification significantly deviate from the gold standard finite element solution, especially at deep indentations (*δ* > 0.1*R*).

Fig. 3 demonstrates that violating the assumptions for the Hertzian contact model leads to significant errors and that modified solutions may correct for some shortcomings but not all. Specifically, the figure shows three surfaces: first, it shows the (gold-standard) finite element solution surface to the indentation problem in red. This surface demonstrates that the indentation force is highly dependent on the size of the indented sample. That is, the thinner the sample, the larger the indentation force. Similarly, the smaller the lateral dimension of the sample, the smaller the indentation force. Additionally, it shows that the deeper the indentation, the larger the indentation force. The figure also shows the Hertzian solution in blue. That is, the Hertzian solution is a good approximation for large and thick samples and small indentations. When these assumptions are violated, the Hertzian solution quickly deviates from the gold-standard solution. In fact, errors may be as large as 89% when the sample becomes as thin as *H* = 2*R*. Finally, the figure also shows the modified Hertzian solution in black. We find that the modified Hertzian solution accurately accounts for increased indentation depth but cannot account for nonlinearities induced by small sample sizes and thicknesses. This is important when applying indentation to biological tissues where lateral sample sizes may be minuscule, and tissue can be very thin^14^

### 3.2 Neural network training & validation

**Fig. 4:**
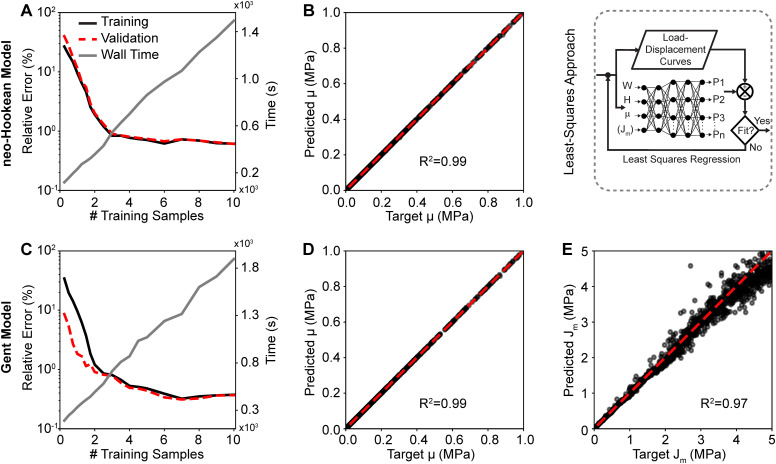
Training and validation of the least squares-based inverse approach. The trained forward neural network for the neo-Hookean model (A) yielded near-perfect agreement between the predicted shear modulus and the ground truth (B). The trained forward neural network for the Gent model (C) also yielded near-perfect agreement between the predicted shear modulus and the ground truth (D), and yielded strong, yet imperfect, agreement between the predicted parameter *J*_m_ and the ground truth (E). Note, comparisons between predicted and target parameters used the neural networks that were trained with 10 000 samples.

Fig. 4 shows the training and validation data for both the neo-Hookean model (top row) and the Gent model (bottom row). Fig. 4(A) and (C) demonstrate that 10 000 samples sufficed to fully train both networks with minimum validation (relative) errors of 0.61% and 0.37% for the neo-Hookean model and Gent model, respectively. The figures also show the linearly increasing cost, *i.e.*, wall time, of increasing training data size. Once we trained the neural network to predict the forward problem, we used the network within a least squares approach to inversely identify the material parameters to the neo-Hookean and the Gent models from synthetic load–displacements curves. Fig. 4(B), (D) and (E) show a comparison between the target parameters *μ* and *J*_m_, with which the synthetic load–displacement curves were created, and the inversely identified – or predicted – parameters for both models. The shown data sets were pulled from the validation pool. Evidently, *μ* of the neo-Hookean model was predicted accurately with a near-perfect correlation of *R*^2^ = 0.99 and an average relative error between the actual and the predicted parameter of 0.26%. Similarly, *μ* of the Gent model was also predicted accurately with a near-perfect correlation of *R*^2^ = 0.99 and an average relative error of 0.58%. However, we found that *J*_m_ was predicted less accurately with an average relative error of 5.22%. Yet, its correlation was still near perfect with *R*^2^ = 0.97 Please note that the error increased with increasing values for *J*_m_.

**Fig. 5:**
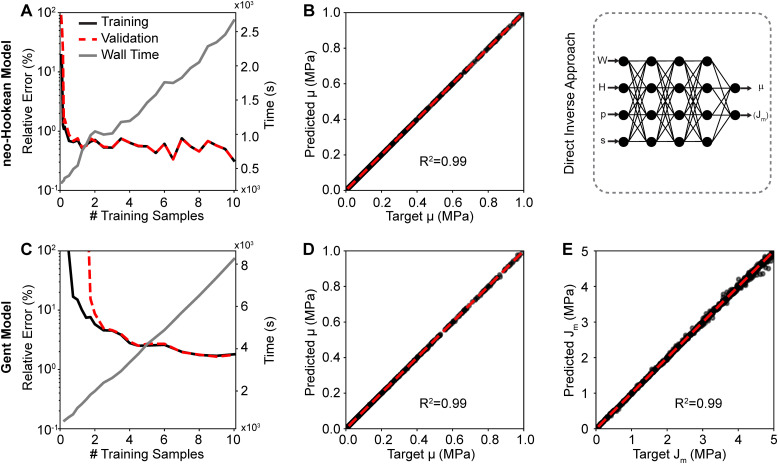
Training and validation of the direct inverse approach. The trained inverse neural network for the neo-Hookean model (A) yielded near-perfect agreement between the predicted shear modulus and the ground truth (B). The trained inverse neural network for the Gent model (C) yielded near-perfect agreement between the predicted shear modulus and the ground truth (D), as well as for the predicted parameter *J*_m_ and the ground truth (E). Note, comparisons between predicted and target parameters used the neural networks that were trained with 10 000 samples.

In our second approach, we trained a neural network to directly map load–displacement curves – as represented through parameters *p* and *s*, see Section 2.2 – to material parameters. Fig. 5 shows the training and validation data of this second approach for both the neo-Hookean and the Gent models. Fig. 5(A) and (C) demonstrate that 10 000 samples sufficed to fully train both networks with minimum validation (relative) errors of 0.70% and 1.77% for the neo-Hookean and Gent models, respectively. Here, again, the figures also show the linearly increasing cost, *i.e.*, wall time, of increasing training data size. To validate the networks, we then applied the direct approach to identify the neo-Hookean and the Gent material parameters from synthetic load–displacement curves. Here, again, we used data from the validation pool. Fig. 5(B), (D) and (E) show a comparison between the target parameters and the predicted parameters for the neo-Hookean and the Gent model, respectively. From these data, it is evident that the shear modulus *μ* of the neo-Hookean model was predicted accurately with an *R*^2^ = 0.99 and an average relative error of 0.68%. Similarly, the shear modulus *μ* of the Gent model was also predicted highly accurately with an *R*^2^ = 0.99 and an average relative error of 0.44%. However, as with the least squares approach, here, too, we found that *J*_m_ was predicted less accurately with an average relative error of 1.38%. Yet, *R*^2^ remained high.

### 3.3 Neural network testing against the Hertzian solution

**Fig. 6:**
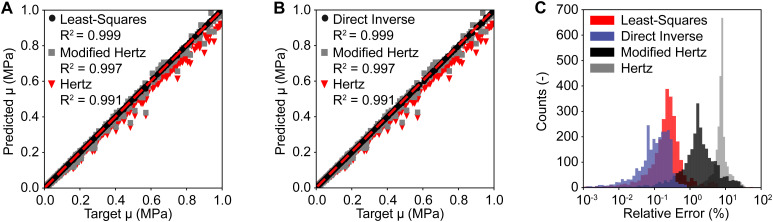
Testing of the least squares and the direct inverse approaches for the neo-Hookean model. Both the least squares approach (A) and the direct inverse approach (B) yield accurate predictions for the shear modulus *μ*. This is especially true when compared to the Hertzian and the modified Hertzian solutions (C) that show significantly higher errors between the predicted and the target shear moduli. Note, these data stem from our testing data pool and were therefore unseen and not used during parameter tuning or model validation.

After training and validating our neural networks for the least squares-based and the direct inverse approach, we tested and compared both approaches against the Hertzian and the modified Hertzian solutions. Fig. 6(A) compares the predicted and the target shear modulus *μ* of the neo-Hookean model between our least squares-based approach, the Hertzian, and the modified Hertzian solutions. Our first approach achieved a low average relative error of 0.60% for the shear modulus *μ* of the neo-Hookean model. In contrast, the Hertzian and the modified Hertzian solutions achieved average relative errors of 8.05% and 3.34%, respectively. Importantly, however, individual errors for both analytical solutions were as high as 100%. Those findings are similar for the direct inverse approach. Fig. 6(B) compares the predicted and the target shear modulus *μ* of the neo-Hookean model between our direct inverse approach, the Hertzian, and the modified Hertzian solutions. Our second approach achieved a low average relative error of 0.69%, compared to the 8.05% and 3.34% reported above. See also Fig. 6(C) for a comparsion between error distrubtions of the Hertzian, the modified Hertzian, and our approaches. Please see ESI,† Fig. S1 and S2 for additional sensitivity analyses, where we study the prediction error as a function of sample geometry, indentation depth, and material stiffness. All approaches were compared using our testing data set that are different from our training and validation data sets used in Fig. 4 and 5.

### 3.4 Neural network testing against real-world data

**Fig. 7:**
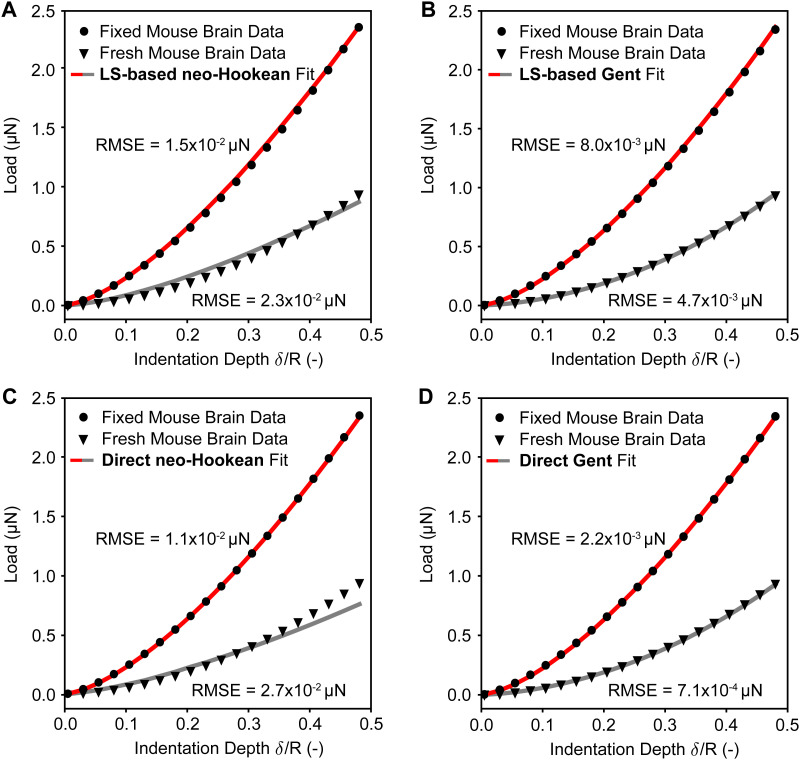
Comparison between the least squares (LS) based inverse approach and the direct inverse approach on real-world indentation data. (A) and (B) Least squares-based fits of the neo-Hookean and the Gent models to both averaged fresh and chemically fixed mouse brain nano-indentation data, respectively. (C) and (D) Fits to the neo-Hookean and the Gent models to both averaged fresh and chemically fixed mouse brain nano-indentation data using the direct inverse approach, respectively. Experimental data was obtained as the average of 686 fresh and fixed individual curves. RMSE = root mean squared error.

**Fig. 8:**
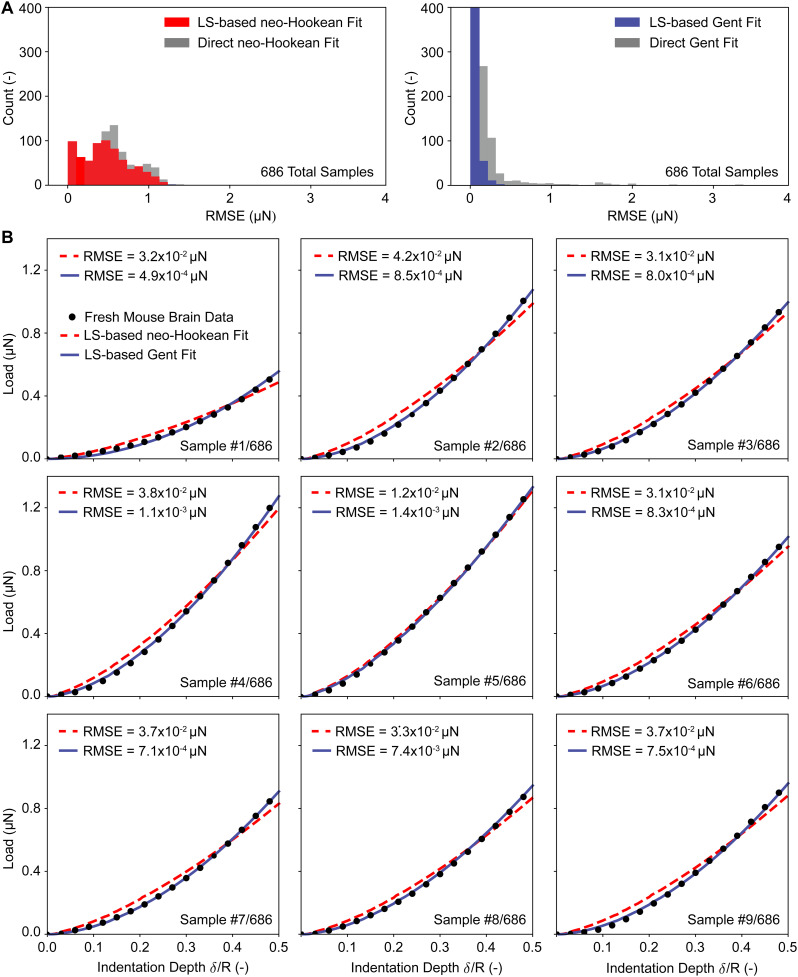
Showcasing our two inverse approaches against real-world data. (A) The error distribution from fitting the neo-Hookean model and the Gent models to 686 fresh mouse brain nano-indentation data sets. We compared both the least squares (LS) based inverse approach and the direct inverse approach. (B) Fits *via* the direct inverse approach to the first nine samples in our data set. RMSE = root mean squared error.

To test both of our approaches against real-world data, we used nano-indentation data of fresh and fixed mouse brains with a total of 1372 load–displacement curves. To this end, we first used our approaches to inversely identify the neo-Hookean and the Gent material parameters from the experimental data. Next, to test the accuracy of our prediction, we used those same parameters in a nonlinear finite element simulation of the nano-indentation problem and compared those predictions to the actual experimental data. In Fig. 7 we compare the load–displacement curves based on our predictions to the average load–displacement data of fresh and fixed mouse brain indentation. Specifically, we first compare our least squares-based approach for both the neo-Hookean and the Gent model in Fig. 8(A) and (B), respectively, before conducting the same comparison for our direct approach in Fig. 8(C) and (D). We find that our predictions fit the experimental data well with root mean squared errors (RMSE) on the order of 10^−4^ to 10^−2^ μN. This is true for both fresh tissue (with lower moduli) and fixed tissue (with higher moduli). We also find that the Gent model fits the experimental data better than the neo-Hookean model, especially for fresh tissue.

Finally, we tested our approach also on all individual load–displacement curves from our fresh tissue data pool. Fig. 8(A) shows the summary statistics of 686 individual inverse analyses and their RMSEs against the experimental data for both the neo-Hookean and the Gent model. These statistics show that the least squares approach yields smaller and narrower distributed errors than the direct approach. Additionally, these statistics reinforce that the Gent model fits the fresh mouse brain indentation data better than the neo-Hookean model. Finally, Fig. 8(B) representatively shows the first nine fits *via* the direct approach for both the neo-Hookean and the Gent model, which reinforces the findings based on our summary statistics.

Also, identifying the material parameter from our mouse brain indentation data set comprised of 1372 samples took 6.85 hours using the least squares approach and 132 seconds using the direct approach. In contrast, using a finite element-based least squares approach to do the same would take approximately 200 days. Note, we extrapolated this number from the cost of a single finite element forward simulation and the same number of iterations as required in our least squares approach. All of these numbers were benchmarked on a personal desktop computer (AMD Ryzen 9 5950X: 16 Cores at 4.9 GHz).

## 4 Open software tool: AI-dente

Our training data, python scripts, trained neural network, and sample data are openly available.‡‡https://github.com/cmsmlab/AI-dente In addition to the synthetic data and the experimental data, our open repository contains the scripts main_SynthData_neoHookean.py and main_SynthData_Gent.py with which the interested reader can simulate the indentation problem using FEBio. The repository also contains the script main_AnalyzeData.py that reads load–displacement data and sample dimensions and outputs the machine learning-based and Hertzian-based predictions for the material shear modulus. Please note, this script also computes Young's modulus and modified Young's modulus as commonly reported in the nano-indentation literature.^46^

## 5 Discussion

In our current work, we implemented a machine learning-based tool to inversely identify material parameters from nano-indentation derived force–displacement data. We set out to overcome (i) the limited accuracy of the Hertzian contact solution for experiments beyond the linear strain regime and (ii) the high computational expense of finite element-based approaches.

### 5.1 Advantage over other approaches

We showed that we successfully accomplished both goals. That is, we showed through two differing approaches that a machine learning-based approach can be both accurate and computationally efficient. First, we trained a neural network to solve the forward indentation problem. By integrating this network into a least squares framework, we could iteratively identify the material parameters of two popular material models, the neo-Hookean model and the Gent model. Additionally, we used a neural network to directly predict those material parameters from load–displacement curves. Both approaches yielded results with errors of <1% and within <1 s, even for very small and thin samples that violate the assumptions of the Hertzian contact solution. The same cannot be said for alternative approaches, such as the inverse finite element approach.

### 5.2 Robustness to experimental noise

We tested our approach against both synthetic data and real-world data. In both cases, we achieved high fit qualities with low errors between predictions and ground truth (in the case of the synthetic data) and between predictions and the experimental data (in the case of real-world data). That is, our approach is robust against experimental uncertainty and noise. Interestingly, the robustness of our approach does not stem from training our networks on synthetic noise. Instead, the robustness of the least squares approach stems from the forward model being effectively constrained through its training to only yield smooth load–displacement curves. On the other hand, the direct inverse approach is highly sensitive to noise when being directly applied to load–displacement data. We overcame this challenge by fitting the synthetic and real-world load–displacement curves to a two-parameter function. Thereby, we effectively parameterized the load–displacement curves akin to a low-pass filter step. Subsequently, we used the two parameters as network input features rather than the potentially noisy raw load–displacement curves.

### 5.3 Material model choice

Albeit not specifically related to our approach but potentially interesting to the reader, we found that the Gent model was the superior model for fitting the strain-stiffening behavior as seen in our real-world data set. Of course, this is hardly surprising as we expect a two-parameter model to outperform a one-parameter model. Nonetheless, we want to highlight the favorable performance of the Gent model. Especially given that the Gent model receives relatively little attention in the biomechanics community and is often forgone in favor of the two-parameter Ogden model, which receives much attention.^47^ One important advantage over its more popular counterpart could be seen in the easier interpretability of its parameters. That is, its parameters are the shear modulus and stiffening parameter. While the Ogden model is also a two-parameter model, its parameters are not as easily associable with physical characteristics. Additionally, the Ogden model suffers from a number of peculiarities that we have recently discussed.^47^ However, it should be noted that the Gent model shows a high degree of nonlinearity in its stiffening parameter. Among other effects, this causes a decrease in its influence with increasing magnitude. For us specifically, this resulted in worse identifiability and increasing training errors for large values of *J*_m_, see Fig. 4 for example.

### 5.4 Limitations

In our work, we limited our training to hyperelastic materials and to a relatively simple contact problem (*e.g.*, we did not account for friction or surface effects, such as surface tension, adhesion, or curvature forces^48,49^). Thus, when more complex contact behavior is required, our tool will not be useful in its current form. However, our framework is generally applicable and could be trained on more complex materials and contact cases. For example, one could train neural networks to learn viscoelastic behavior of soft tissue and to learn surface effects between indenter and sample.^50^ Thus, not only is our framework accurate and fast, but it is also highly flexible. Of course, additional training requires additional synthetic data, as well as new validation, and testing. Thus, the interested reader/user would have to weight the cost of extending our framework against the cost of conducting instead an inverse finite element analysis.

## 6 Conclusion

We proposed and successfully tested a machine learning-based approach to determine material parameters from nano-indenter-based load–displacement curves. That is, we showed that we can use machine learning to yield accurate and fast results that outperform both the classic Hertzian solution (especially for very small and thin samples) and a traditional finite element-based approach. In addition to being accurate and fast, our approach is also highly flexible and allows accounting for complex material behaviors and nonlinear contact phenomena.

## Conflicts of interest

Dr. Manuel K. Rausch has a speaking agreement with Edwards Lifesciences. No other author has conflicting interests to report.

## Supplementary Material

SM-019-D3SM00402C-s001
