# Supplementary material for: AI-dente: an open machine learning based tool to interpret nano-indentation data of soft tissues and materials
Source: Soft Matter. 2023 Aug 25;19(35):6710–20. doi: 10.1039/d3sm00402c (PMC10499084; doi:10.1039/d3sm00402c)
Supplement: SM-019-D3SM00402C-s001 [file SM-019-D3SM00402C-s001.pdf]

Electronic Supplementary Material (ESI) for Soft Matter.
This journal is © The Royal Society of Chemistry 2023

SUPPLEMENTARY INFORMATION

AI-dente: An Open Machine Learning Based Tool to Interpret Nano-indentation Data of Soft Tissues and Materials

Giolando et al.

The University of Texas at Austin

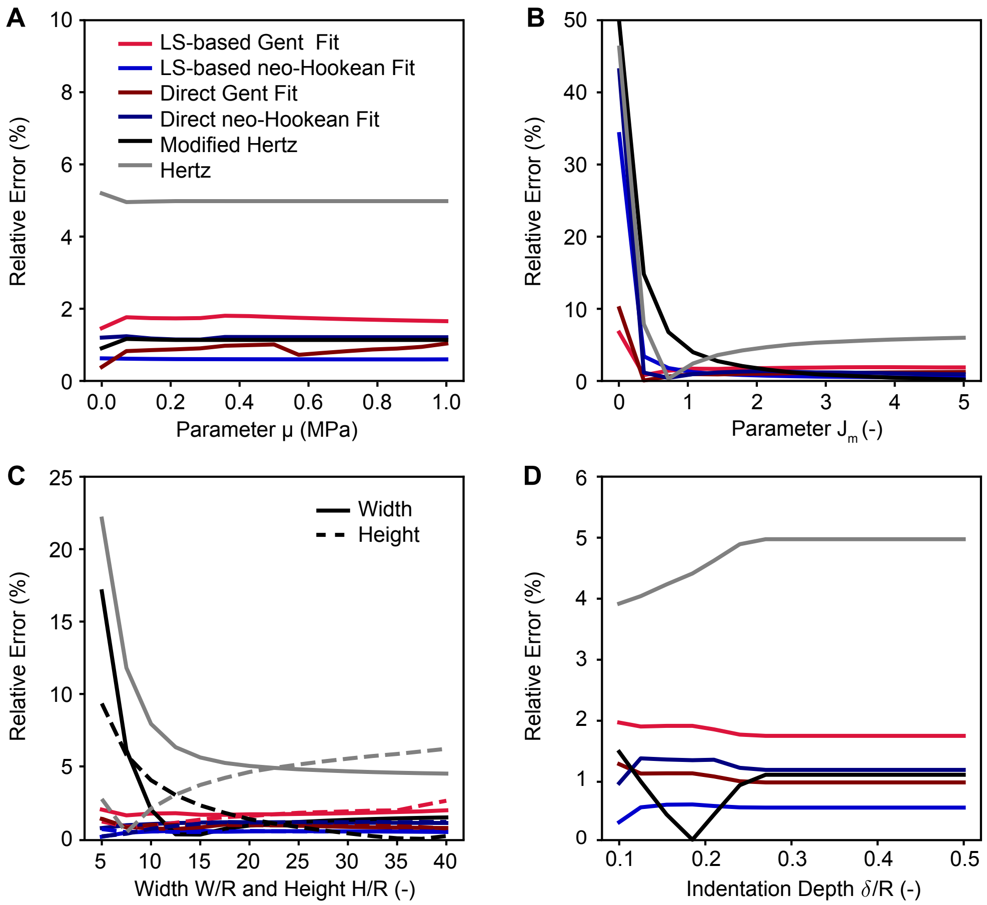

Figure S1: Sensitivity of shear modulus identifiability for all methods. The error between the predicted and the ground truth shear modulus as a function of A) sample shear modulus, B) stiffening parameter, C) sample width and height, and D) indentation depth.

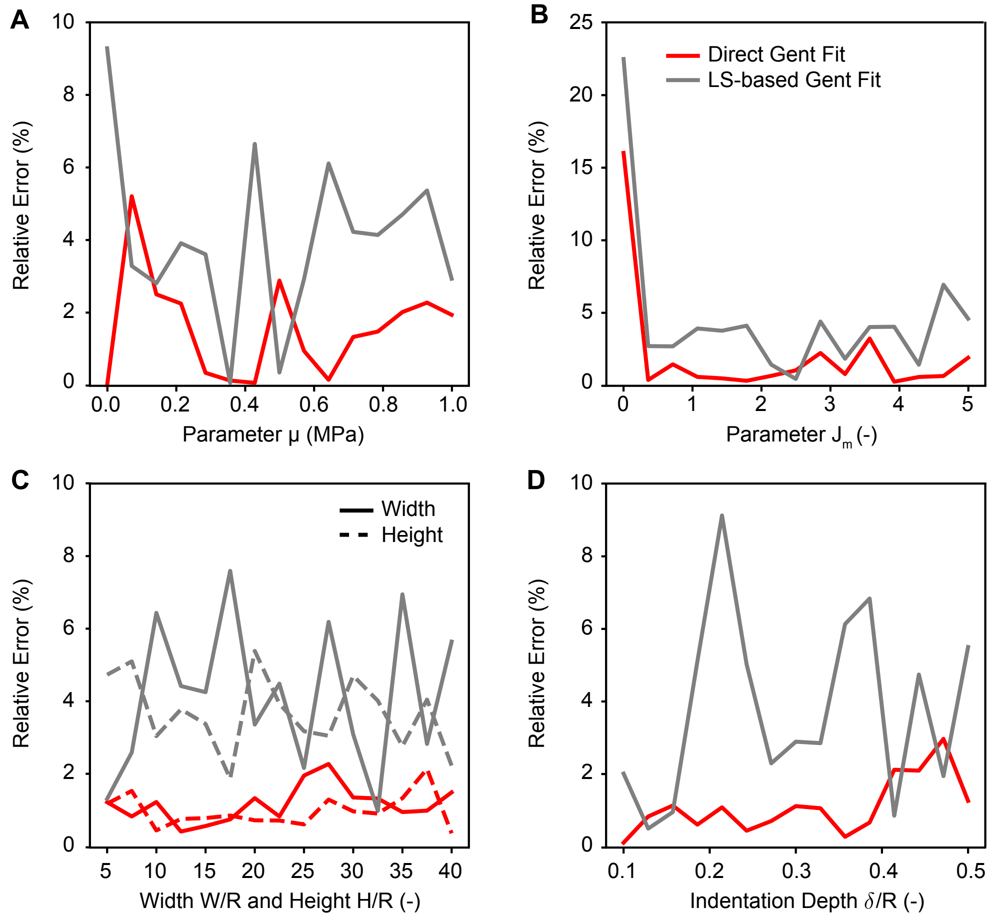

Figure S2: Sensitivity of stiffening parameter J_m identifiability for all methods. The error between the predicted and the ground truth stiffening parameter as a function of A) sample shear modulus, B) stiffening parameter, C) sample width and height, and D) indentation depth.
